# Catalysts for Hydrogen Generation via Oxy–Steam Reforming of Methanol Process

**DOI:** 10.3390/ma13245601

**Published:** 2020-12-08

**Authors:** Magdalena Mosińska, Małgorzata I. Szynkowska-Jóźwik, Paweł Mierczyński

**Affiliations:** Institute of General and Ecological Chemistry, Lodz University of Technology, Zeromskiego 116, 90 – 924 Lodz, Poland; magdalena.mosinska@dokt.p.lodz.pl (M.M.); malgorzata.szynkowska@p.lodz.pl (M.I.S.-J.)

**Keywords:** hydrogen production, oxy–steam-reforming of methanol, heterogeneous catalysts, surface reaction mechanism

## Abstract

The production of pure hydrogen is one of the most important problems of the modern chemical industry. While high volume production of hydrogen is well under control, finding a cheap method of hydrogen production for small, mobile, or his receivers, such as fuel cells or hybrid cars, is still a problem. Potentially, a promising method for the generation of hydrogen can be oxy–steam-reforming of methanol process. It is a process that takes place at relatively low temperature and atmospheric pressure, which makes it possible to generate hydrogen directly where it is needed. It is a process that takes place at relatively low temperature and atmospheric pressure, which makes it possible to generate hydrogen directly where it is needed. This paper summarizes the current state of knowledge on the catalysts used for the production of hydrogen in the process of the oxy–steam-reforming of methanol (OSRM). The development of innovative energy generation technologies has intensified research related to the design of new catalysts that can be used in methanol-reforming reactions. This review shows the different pathways of the methanol-reforming reaction. The paper presents a comparison of commonly used copper-based catalysts with other catalytic systems for the production of H_2_ via OSRM reaction. The surface mechanism of the oxy–steam-reforming of methanol and the kinetic model of the OSRM process are discussed.

## 1. Introduction

**Figure 1:**
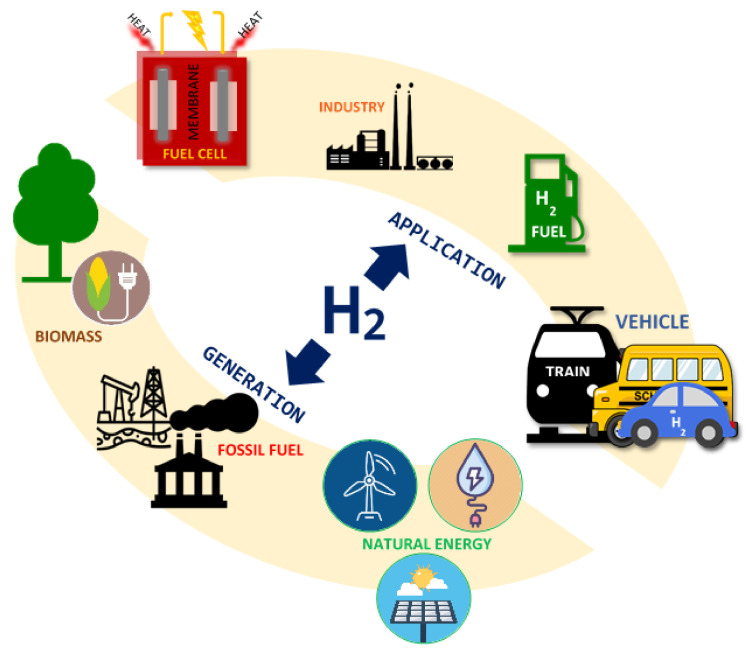
Hydrogen as an energy carrier.

The economic and civilization development of the world has caused the population and the total energy consumption to have increased significantly recently. The current state of the natural environment and the shrinking reserves of crude oil, resulting from excessive consumption of fossil fuels, have intensified research related to the use of alternative fuels and energy generation technologies [1]. The use of fossil fuels has a negative impact on the environment and causes the emission of harmful oxides into the atmosphere. The emission of an excessive amount of harmful gases into the atmosphere is responsible for the formation of smog and the greenhouse effect. This is a serious problem for animals, plants and human health [2]. In addition, it should be emphasized that fossil fuel sources are not renewable. Their continuous exploitation may finally lead to the exhaustion of their sources. The greatest challenge nowadays is the production of energy from renewable sources. One of the promising alternatives to fossil fuels is hydrogen [3]. Hydrogen is a pro-environmental energy carrier. In the burning flame arise only water vapor and nitrogen oxides, and when the oxidation process is carried out in fuel cells, the only byproduct is water vapor. Hydrogen also has a low ignition energy of initiation, which improves combustion. It is worth noting that the sources of hydrogen are practically inexhaustible. The increased interest in hydrogen is also associated with its usage to power fuel cells as one of the possible alternatives to replace fossil fuels [4,5]. Current research proves that the use of hydrogen to power fuel cells is one of the most effective and environmentally friendly technologies of transforming chemical energy into electricity, which does not generate additional pollution [5,6]. Hydrogen and fuel cell are a great opportunity to reduce emissions of compounds such as nitrogen oxides, carbon oxides, various hydrocarbons, which cause acid rain and possibly the greenhouse effect. The utilization of hydrogen and fuel cells can eliminate the formation of smog in highly urbanized cities. In addition, due to the fact that hydrogen used to power fuel cells should be highly pure, because small amounts of carbon monoxide (above 10 ppm) in the gas supplying the fuel cell cause its irreversible adsorption and poisoning of platinum electrodes used in fuel cells [7]. Therefore, the key task seems to be the development of a method or technology for the production of high purity hydrogen. The production of pure hydrogen is an important problem in the modern chemical industry. However, the industrial production of hydrogen-based on natural gas reforming is well known; it is still a problem to find low-cost methods for obtaining hydrogen intended for supplying small or mobile devices, such as fuel cells. High efficiency, durability and reliability of fuel cell technology represents a major potential, pointing to the possibility of the rapid development of industries that use fuel cells. The possible ways of hydrogen generation and application are presented in Figure 1. There are many sources of hydrogen that can include hydrocarbons, alcohols, ammonia and others. However, methanol is one of the most promising sources of hydrogen because it is the simplest alcohol without a C–C bond in the molecule and provides a high H:C ratio in a molecule. Its properties indicate that methanol can be easily reformable in the low-temperature range and under atmospheric pressure. Considering the relatively mild conditions of the methanol-reforming process (*p* = 1 atm, T = 160–350 °C), it is possible to generate hydrogen practically everywhere. Production of hydrogen through mixed oxy–steam-reforming of methanol enables to be carried out the process autothermally, which is extremely beneficial from the economics point of view.

**Figure 2:**
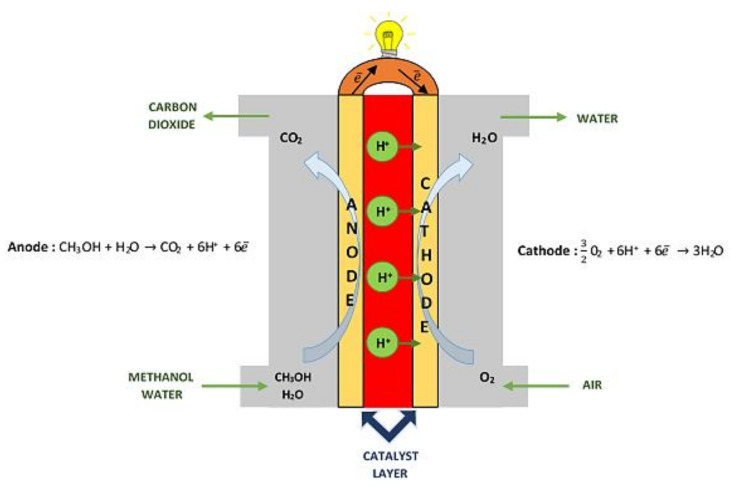
Direct methanol fuel cells (DMFC).

Methanol fuel cell DMFC (direct methanol fuel cell) is particularly promising. The choice of methanol as a fuel is dictated by the high energy density of methanol and relatively easy oxidation of methanol at a relatively low temperature of 40–80 °C. The principle of operation of a methanol-powered fuel cell is shown in Figure 2. One of the most important applications of fuel cell technology is hybrid systems, which are widely used in the first commercial portable power generators and prototype electric vehicles [8]. Portable power generators are based on a methanol fuel cell (direct or generators equipped with a micro-reformer) [9] and lithium–ion battery. Prototype mobile vehicles use nickel-hydride battery used for starting purposes or for maintaining stable engine operation. These vehicles achieve an efficiency of about 50%, which is a value higher than in hybrid cars equipped with internal combustion engines. The disadvantage of these systems is their present cost, which is difficult to reduce [9]. The basic condition that hydrogen could replace current energy sources is to develop a cheap, efficient and rapid method for hydrogen production.

The importance of hydrogen as a fuel in the twenty-first century still increases with the further development of fuel cells, which are used in automotive, power generation, power supplies, cell phones and laptops. Due to the above reasons, one of the most important areas of the research in the field of hydrogen production, which are undertaken by scientific groups around the world, is the design of new catalytic systems, which must be characterized by high activity and selectivity in the reforming of methanol reaction. An important aspect of the developed catalysts is their high stability, which they should have in order to be used to power fuel cells applied in mobile vehicles or stationary systems constituting an emergency energy source in public places (including military bases, offices, hospitals, administrative buildings, etc.). Their applicability increases due to their quiet operation, the quality of the supplied energy, the possibility to transport and application practically everywhere.

## 2. Methanol Reforming Reactions

There are many possible sources of hydrogen that can include hydrocarbons, alcohols, ammonia and others [5,10,11,12,13,14,15,16]. An attractive alternative solution to problems associated with storing molecular hydrogen involves on board catalytic production of hydrogen from a high energy liquid fuel such as methanol. Methanol is one of the most promising sources of hydrogen because it is the simplest alcohol without a C–C bond in the molecule and provides a high H: C ratio in a molecule. Its properties indicate that methanol can be easily decomposed to a hydrogen-rich mixture practically everywhere. The hydrogen can be synthesized from the catalytic reforming of methanol, decomposition of methanol, partial oxidation of methanol, or a combination of two processes into one reaction, which is named the oxy–steam-reforming of methanol [4,17,18,19,20,21]. Thermodynamics of the processes of partial oxidation and steam-reforming of methanol suggests that the most energetically favorable solution is a combination of these two reactions in one process. Depending on the stoichiometry of the reaction between methanol, water vapor and oxygen, the methanol processing reactions run according to the following equations:

Steam reforming of methanol (SRM)

CH_3_OH + H_2_O → CO_2_ + 3 H_2_ ΔH° = 49.7 kJ/mol (1)

Decomposition of methanol (MD)

CH_3_OH → CO + 2 H_2_ ΔH° = 128.5 kJ/mol (2)

Partial oxidation of methanol (POM)

CH_3_OH + 0.5 O_2_ → CO_2_ + 2 H_2_ ΔH° = −192.2 kJ/mol (3)

Oxy–Steam Reforming of Methanol (OSRM—a combination of SRM and POM)

CH_3_OH + 0.5 H_2_O + 0.25 O_2_ → CO_2_ + 2.5 H_2_ ΔH° = −71.4 kJ/mol (4)

It is worth emphasizing that the most energetically beneficial process is oxy–steam-reforming of methanol being a combination of steam and partial oxidation of methanol in one process. In addition, this process can be carried out at low temperature (160–350 °C) at atmospheric pressure without the formation of carbon deposits [18,22,23]. This means that hydrogen can be directly produced where it is needed. The production of hydrogen by the oxy–steam-reforming methanol method allows the reaction to be carried out in an auto-thermal way, which is very beneficial from an economic point of view. The supported catalysts used in this reaction undergo deactivation process due to carbon deposition or as a result of overheating. The problem is obtaining pure hydrogen without carbon monoxide. CO is a strong poison of platinum catalyst used directly in fuel cells.

**Figure 3:**
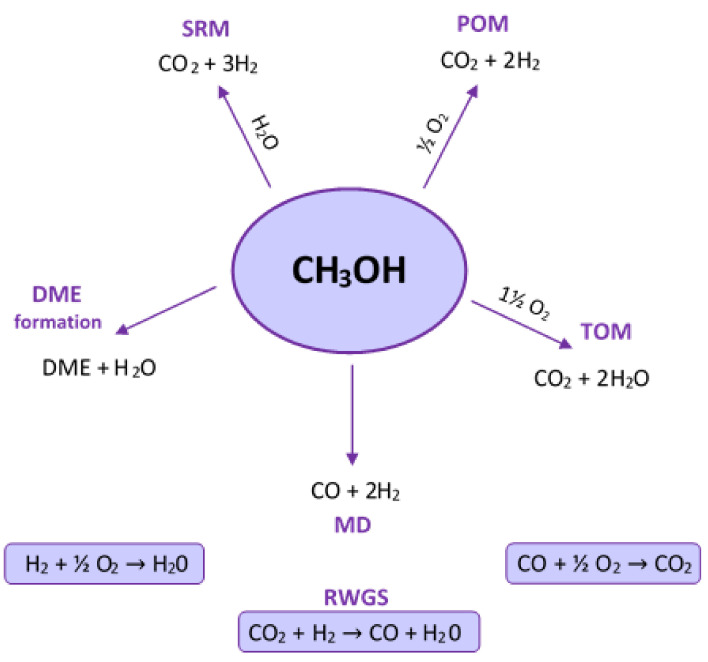
The possible reactions for the autothermal reforming of methanol process [24].

Geissler et al. [24] developed the kinetic model for the autothermal reforming of methanol reaction over CuO/ZnO/Al_2_O_3_ catalyst. They reported that the eight possible reactions take place during the autothermal reforming of the methanol process, and seven components were present in the feed or product gases of the reaction (see Figure 3). The reaction scheme showed that the reactions strongly depend on each other because most of the components occur in more than one reaction. In addition, the products of one reaction can be reactants in other reactions. All reactions which run in the presence of oxygen are very fast and strongly exothermic compared to the reverse water gas shift (RWGS) process. This result proves that the major source of hydrogen is the steam-reforming of methanol reaction. Moreover, the dimethyl ether (DME) formation can be considered independently from the other reactions, which runs parallel with another autothermal reforming of methanol reactions. The reactor to autothermal reforming of methanol process is divided into two parts. Strongly exothermic reactions with oxygen take place at the entrance to the reactor. In the lower parts of the catalyst bed, the endothermic reactions, including mainly steam-reforming of methanol, are carried out. Furthermore, two models of autothermal reforming reactions (ATRM) can be considered, named partial oxidation–steam-reforming of methanol (POM-SRM) and total oxidation–steam-reforming of methanol (TOM-SRM). The coefficients describing the number of reactants, which must be taken for the mentioned processes of the methanol processing, are shown in Table 1. The experimental analysis clearly showed that in the presence of oxygen, the methanol conversion occurs mainly via the total oxidation (TOM) reaction. However, the hydrogen production is subsequent to the steam-reforming (SRM) reaction.

## 3. Surface Reaction Mechanism and Kinetic Models of the Oxy–Steam Reforming of Methanol Process on Copper-Based System

The literature review concerning the catalysts used in the steam, partial oxidation and oxy–steam-reforming of methanol reactions shows that the typical catalysts of the discussed processes are copper catalysts supported on both monoxides: Al_2_O_3_, ZnO, CeO_2_, MgO, La_2_O_3_, SiO_2_, and bi-oxides: ZnO–Al_2_O_3,_ CeO_2_–ZrO_2_, SiO_2_–SnO_2_, Al_2_O_3_–CeO_2_ [18,25,26,27,28,29,30,31,32,33,34,35,36,37,38]. Metallic copper surface determined by the chemisorption method, high values of copper dispersion is the targets to attain for the achievement of highly active catalysts. In the literature data, there are many works investigating the addition of promoters [30,32,39,40] and the influence of the preparation method [41,42,43] on catalytic properties of copper supported catalysts. Despite the high activity and selectivity of copper catalysts in the methanol conversion reaction, research is still ongoing to improve their catalytic properties in the investigated process [6]. Therefore, many research centers are trying to understand the mechanism and kinetics of individual methanol processing reactions [44,45,46,47,48,49,50,51,52]. The mechanism of methanol processing reactions has been studied in the literature data by various scientists, but H_2_ production from methanol is an issue still raised in many works. Various mechanisms of methanol reforming processes have been proposed in the literature, depending on the role of various active centers present on the copper catalyst surface, such as Cu^0^ [53], Cu^+^ [54] or Cu^0^–Cu^+^ [49] couples. The methanol adsorption and decomposition measurements performed on Cu100 and Cu110 surfaces showed that methanol dissociates from methoxy species (CH_3_O). The recent studies proved that the presence of absorbed O on the copper surface enhances the formation of methoxy species [49]. Other authors also reported that methanol interacted very weakly on clean Cu(100) [55], Cu(110) [56], and Cu(111) [57] surface, what confirmed the need for activation of the catalysts by partial exposure of the catalyst surface by O_2_. Wachs et al. [56] reported that the dissociative chemisorption of CH_3_O-H on Cu increased by the specific interaction, which takes place between the hydroxyl end of the methanol molecule and surface oxygen atoms (see Table 3). Moreover, the Cu^δ+^O^δ-^ site facilitates the breakdown of the O-H bond present in the methanol molecule. The surface oxygen comes from the incomplete reduction of the catalyst or reaction atmosphere formed near the space of the catalyst bed during the reaction. In addition, Fisher et al. [56] reported about the dissociation of water to H_2_ and O even at 73 °C on reduced polycrystalline Cu [58]. The reaction scheme of POM, SRM and OSRM reactions is complicated by a few secondary reactions which run in parallel to the methanol dehydrogenation process. These reactions are listed below:

Total oxidation of methanol (TOM)

CH_3_OH + 3/2O_2_ → 2CO_2_ + 2H_2_ (5)

Water-gas shift reaction (WGS)

(6) $$\mathrm{CO}\;+\;H_{2}O\;⇄H_{2}+{\mathrm{CO}}_{2}$$

PROX reaction

(7) $$\mathrm{CO}\;+\;1/2O_{2}\;⇄{\mathrm{CO}}_{2}$$

Oxidation of H_2_

(8) $$H_{2}\;+\;1/2O_{2}\;⇄H_{2}O$$

**Figure 4:**
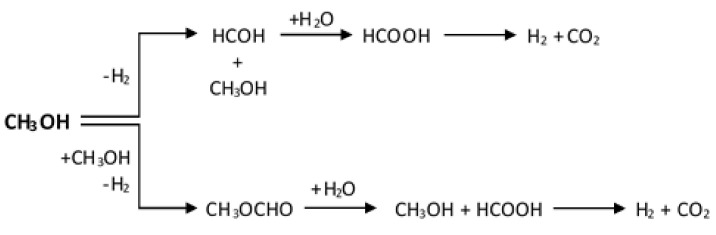
The reaction steps taking place during the steam-reforming of methanol process [69].

Taking into consideration the above processes, it could be emphasized that CO could be produced or reacted within all presented above reactions. That is why the concentration of carbon monoxide should be carefully controlled, taking into account the further use of reforming of methanol reactions for the production of hydrogen for fuel cells. It should be remembered that CO is a serious poison of platinum electrodes used in fuel cell technology. It is well known that even trace amounts of carbon monoxide can chemisorb irreversibly on the surface of the platinum electrodes, stopping the operation of the fuel cell. Methanol decomposition has been regarded as the easiest process of H_2_ production from methanol, and it runs during other methanol-reforming processes, including SRM, OSRM and POM. Up to date, there are four reaction possible schemes proposed for SRM reaction. Namely, MD-WGSR scheme, 1-step SRM scheme, SRM-MD-reverse-WGSR scheme and methyl formate scheme [59] (see Table 3). According to the MD-WGSR reaction scheme, CO is the primary product of the reaction, which is produced from the methanol dehydrogenation process. Then in the next step, CO subsequently is converted into CO_2_ via water gas shift (WGS) reaction [60]. Within the 1-step of the SRM reaction, CO_2_ and H_2_ are formed directly from the methanol dehydrogenation process [24]. This 1-step SRM scheme may be complicated in the case of the reaction carried out with high methanol conversion and contact time where CO started to be formed. Breen et al. [61] investigated the Cu/ZnO/Al_2_O_3_ catalysts promoted by ZrO_2_ and reported that the conversion of methanol was completed at about 345 °C and CO has formed starts from 300 °C, even though its formation is thermodynamically permitted at a lower temperature. In addition, the concentration of CO increase with increase of the reaction temperature and is not formed at low contact time. The reaction conditions of the process are presented in Table 3. Agrell et al. [62] reported that the low CO concentration is connected with the fact that CO is formed during the reverse-WGSR process, which runs during the reforming process. The authors also have experimentally confirmed that the quantity of CO formed during the process was decreasing with reducing contact time. This result indicates that a short contact time decreases the quantity of CO produced from the reverse-WGSR reaction. Peppley et al. [45] also investigated the SRM reaction and claimed that MD-WGSR and 1-step SRM schemes did not describe the mechanism of this reaction. Based on their results, they reported that all the MD, WGSR and SRM steps must be included in the mechanism model of the investigated reaction. The authors claimed that during SRM, MD process run parallel as a side reaction. The products of steam-reforming of methanol process are consumed via reverse-WGSR process (see Table 3). The kinetic model suggested by the authors [63,64,65] confirmed that two different centers present on the catalyst surface were required. One kind of the active centers are required for both the SRM and reverse WGSR processes (see Table 3). The second kinds of the active centers are required for the methanol decomposition reaction. On the other hand, a methyl formate scheme of steam-reforming of methanol has been also proposed by several scientists [66,67,68] (see Table 3). According to this scheme at first step methanol is dehydrogenate to methyl formate and then, the methyl formate is hydrolyzed into formic acid. In the next stage of the process the formic acid decompose to CO_2_ and H_2_ which were the primary products of the system. All of the steam-reforming of methanol reaction stages remaining above are presented in Figure 4.

Authors based on their results reported that methanol dehydrogenation reaction is the rate-determining step of the SRM process. However, the MD-WGSR scheme could be ruled out because the concentration of CO in the product mixture was below the predicted value resulting from the equilibrium of the SRM process. The produced CO via SRM reaction is a primary product, which was produced by the decomposition of methyl formate according to the reaction below:

HCOOCH_3_ → CH_3_OH + CO (9)

Partial oxidation of methanol reaction carried out using molar ratios of O_2_/CH_3_OH equal 0.3 and 0.4 were tested by Murcia-Mascaros et al. [70] (see Table 3). The catalytic activity results obtained by the authors indicated that both methanol conversion and concentrations of products strongly depend on the O_2_ concentration in the reaction mixture. During the partial oxidation of methanol, a significant amount of water and CO_2_ together with a small amount of H_2_ was observed at low methanol conversion and in the case of the reaction during which O_2_ was not completely consumed. Therefore, TOM and MD processes were proposed to be the dominating reactions at the initial stage of the partial oxidation of methanol. In contrast, when oxygen in the reaction mixture is almost consumed, the conversion of methanol increased to a value higher than that resulting from the reaction stoichiometry. At the same time, selectivity towards H_2_ production increases and towards CO formation decreases. Based on the obtained results, the authors reported that during the TOM process, a parallel SRM reaction takes place. Agrell et al. [11] also proposed a scheme of TOM-SRM consecutive pathway reactions for the partial oxidation of methanol reaction. Rabe and Vogel [71] also investigated POM reaction on Cu/ZnO/Al_2_O_3_ catalyst using thermogravimetric analysis coupled with Fourier-transform infrared (FTIR) spectroscopy (see Table 3). The authors reported that the POM reaction from an oxygen-poor mixture leads to the formation of formaldehyde and water. The results also confirmed that CO_2_ was produced as a primary product of the POM reaction. According to the literature [11], the OSRM reaction is very similar to the partial oxidation of the methanol reaction scheme, in which TOM runs successively through the SRM. The reaction scheme ends with a partial CO oxidation or RWGS reaction. The hydrogen and water production via the OSRM process is related to the relation between copper species and the TOM-SRM consecutive reaction scheme. During the TOM reaction, the metallic copper surface of the pre-reduced Cu/ZnO/Al_2_O_3_ catalyst was completely oxidized into Cu^2+^ by the oxygen present in the reaction mixture. At the point at which oxygen is consumed in the subsequent reaction SRM, the reaction mixture may again become a reduction. As a result, Cu^2+^ can be converted back into metallic copper. In addition, the copper species play different roles during OSRM depending on the degree of oxidation of Cu. Cu^2+^ species show negligible activity in H_2_ production. These centers are active in the formation of water and carbon dioxide. Whereas, metallic copper are very active for the H_2_ production. The above proposed mechanism is compatible with the results reported by Reitz et al. [53]. They studied the OSRM reaction using Cu/ZnO catalyst by X-ray absorption near edge structure (XANES) technique. The results showed that at low methanol conversion value, Cu^2+^ was the dominant Cu species, and combustion was the main reaction. In the case of the total conversion of O_2_, Cu^2+^ species were reduced to Cu^0,^ and as a consequence, H_2_ is produced via the SRM process. Agrell et al. [11] reported that the gaseous products formed in POM and OSRM reactions and methanol conversions value were very similar. At the same time, during the SRM reaction, both the methanol conversion and H_2_ production were initiated at a lower reaction temperature on the metallic copper surface. Whereas, during POM and OSRM reactions the methanol conversion and H_2_ production were slower at a higher reaction temperature. The lower catalytic activity of both POM and OSRM reactions should be related to TOM process occurred on the Cu^2+^ species. Whereas, the higher catalytic efficiency is attributed to the SRM reaction that takes place on the metallic copper surface. On the other hand, Patel and Pant [72] proposed a different reaction scheme for OSRM reaction. The conditions of the OSRM process are given in Table 3. Authors reported that during OSRM reaction, partial oxidation of methanol, steam-reforming of methanol and RWGS processes run parallel. A kinetic model for the OSRM system over Cu/ZnO/CeO_2_/Al_2_O_3_ catalyst was developed by using Langmuir–Hinshelwood mechanism. The authors based on their results proposed that TOM was impossible due to the low molar ratio of O_2_ to CH_3_OH in a reaction mixture together with the excess of steam introduced in a reaction mixture used during the oxy–steam of methanol reaction. The scientists claimed that there are two different types of active sites on the catalyst surface. First kind of the active centers are used for the adsorption of C- and O-containing species. While, the second types of the active centers present on the catalyst surface are designed for the adsorption of H. According to the previous work describing in the literature data [73,74,75] (see Table 3), CO is formed as a secondary product from the consecutive RWGS process. The kinetic model proposed by the authors is based on the assumption that formate is formed from oxymethylene in the process of partial oxidation of methanol, which determines the reaction rate determining step (RDS). In this model steam-reforming of methanol run through the dissociation of formic acid, from which adsorbed carbon monoxide and hydroxyl groups are formed. The predicted mechanism was also proved by the experimental data obtained by the authors in the OSRM process. Turco et al. [49] also determined the kinetic parameters based on the methanol conversion values in order to understand the differences in the activity of the investigated catalytic systems (see Table 3). They calculated the kinetic constant k (s^−1^ cm^3^ gcat^−1^) from the equation presented below and assumed for OSRM reaction that fractional expansion *ɛ* = 0.32.

(10) $$k=\frac{1}{\tau }\left[\varepsilon x+\left(1+\varepsilon \right)ln\left(1-x\right)\right]$$

where, τ—contact time (s∙cm^−3^ ∙g_cat_) and *x*—fractional conversion;

Kinetic studies for the OSRM process based on copper catalysts have been conducted. Moreover, the Arrhenius plots presented by the authors show a satisfactory linear regression. Table 2 presents the activation energy values (Ea) obtained for the investigated catalysts. It was also observed that the k values for the OSRM process are not correlated with the copper area. This result is a consequence of the complexity of the OSRM reaction. During the OSRM process, the POM reaction takes place, which is catalyzed by copper oxide in the first zone of the catalyst bed with access to high oxygen concentration. They reported that the interaction with other oxides could influence the activity of such Cu oxide, which may have an effect on the kinetics of POM reaction.

**Figure 5:**
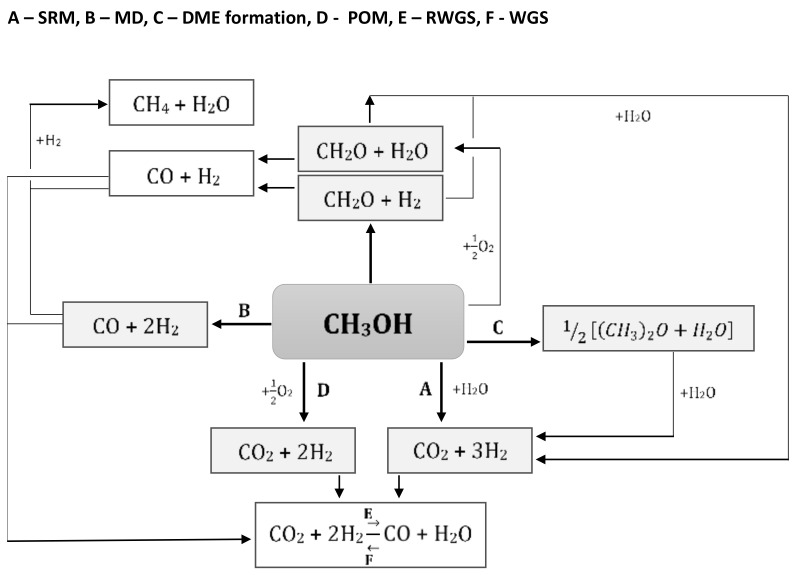
The reaction network taking place during oxidative steam-reforming of methanol (OSRM) process [48].

Turco et al. [48] also investigated the OSRM mechanism on Cu/ZnO/Al_2_O_3_ by FTIR technique, and they reported in their work that methanol adsorbs dissociatively on the catalyst surface (see Table 3). Methoxy groups are adsorbed both on the active sites and on inert regions of the catalyst surface, mainly associated with alumina. Methoxy groups in the vicinity of copper or adsorbed directly on it are easily transformed into surface forms of the formate. Based on their research, the authors discovered traces of formaldehyde also adsorbed on the catalyst surface. In the next step, the formate groups decompose to CO at or above 300 °C. The FTIR measurements also confirmed the formation of dimethyl ether during the OSRM process. Base on the above discussion, the authors proposed the following reaction scheme of the OSRM process carried out on the copper catalyst supported on ZnO·Al_2_O_3_ carrier (see Figure 5).

**Figure 6:**
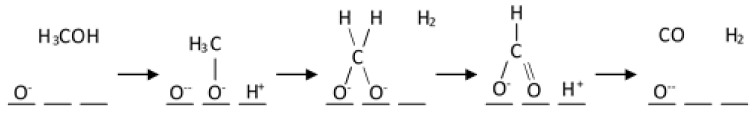
The methanol decomposition (MD) mechanism [60].

The authors reported in their work that the mechanisms of MD (reaction B), POM (reaction D), and SRM (reaction A) reactions are closely related and that the surface phenomena are similar. They also observed that the conversion of methanol in the presence of both water (SRM) and oxygen (POM) is higher compared to the methanol decomposition reaction what is directly related to the oxidation state of Cu present on the catalyst surface. It should also be emphasized that a small amount of carbon monoxide is formed in the presence of water (SRM), and CO_2_ is the dominant product. The FTIR results clearly showed that CO and CO_2_ are formed in appreciable amounts in the POM reaction. It is also known that CO is adsorbed very weakly on metallic copper particles [52], while it is adsorbed very strongly on Cu(I) centers. CO is easily oxidized to CO_2_ on Cu(II) centers. During the methanol decomposition reaction, the catalyst is completely reduced, and in this case, CO is produced by the decomposition of the formate groups. In contrast, the catalyst is partially oxidized during the POM reaction. Therefore, the Cu(I) sites present on the catalyst surface may strongly adsorb CO, while Cu(II) allows its oxidation to CO_2_ before its desorption. Based on this assumption, CO_2_ is the main product of the POM process. During the POM reaction, when O_2_ is consumed, the excess methanol partially decomposes to CO. However, in the case of the SRM reaction, CO is formed in a limited amount. It can also be assumed that the water vapor present during the SRM process oxidizes the catalyst surface, leading to the formation of Cu (II) centers on which CO_2_ is formed. The authors also report that the scheme of the methanol decomposition mechanism was reasonably proposed by Riva et al. [76] (see Figure 6).

## 4. Catalysts Configuration Systems Applied for Hydrogen Production in the Oxy–Steam Reforming of Methanol Process

A literature review concerning catalysts systems applying in reforming of methanol processes indicates that the typical catalysts are Cu, Ni, Co, Fe, Pd, Pt, Ru, Au, Ir, Ag supported on mono-Al_2_O_3_, ZnO, CeO_2_, MgO, La_2_O_3_, SiO_2_ [21,73,78,79], and binary oxides: ZnO-Al_2_O_3_, CeO_2_-ZrO_2_. SiO_2_-SnO_2_, A1_2_O_3_-CeO_2_ [32,73,79,80]. Those catalytic systems are prepared using different methods including: microemulsion [81], aerogel [82], co-precipitation [43], sol–gel [83], impregnation [46], combustion synthesis [84], and others. However, still, the most common catalysts used for oxy–steam reforming of methanol reaction are the copper-based systems. Their metal surface area is determined by the chemisorption method; high values of copper dispersion are the targets to attain for the achievement of highly active catalysts. In the literature data, there are many works investigating the addition of promoters [30,32,39,40] and the influence of the preparation method [41,42,43] on catalytic properties of copper supported catalysts. Despite the high activity and selectivity of copper catalysts in the methanol conversion reaction, research is still ongoing to improve their catalytic properties in the investigated process [6].

### 4.1. The Influence of the Preparation Method on the Catalytic Properties of the Tested Catalytic Systems in the Oxy–Steam Reforming of Methanol Process

The effect of the preparation method of copper catalysts on their catalytic properties in the oxidative steam-reforming of methanol was studied by Shen and Song [42]. They have compared the physicochemical properties of copper catalysts synthesized by impregnation, co-precipitation and hydrothermal synthesis methods, and they claimed that the systems prepared using the co-precipitation method exhibited higher surface area (46% higher than wet impregnation) and methanol conversion in the oxidative steam-reforming of methanol reaction. The activation of the catalyst through the reduction process performed before each catalyst test at lower temperature results in increasing the activity towards hydrogen production. The CuO/ZnO/Al_2_O_3_ catalyst prepared by the co-impregnation method was also examined in the oxidative steam-reforming of the methanol process. The lowest CO concentration close to zero was observed when H_2_O/methanol and O_2_/methanol ratio equal to 1.43 and 0.47, respectively. Papavasiliou et al. [39] investigated Cu-Mn spinel oxide catalysts as an alternative to a commercial CuO/ZnO/Al_2_O_3_ catalyst. The physicochemical properties and catalytic characterization of the investigated catalysts applied in OSR of methanol reaction are given in Table 4. The Cu_x_-Mn_y_ catalytic systems were synthesized by the urea nitrate combustion method. The activity results showed that the investigated catalysts exhibited high activity to H_2_ production in combined (oxy–steam) reforming of methanol reaction. The most active catalysts which exhibited almost identical activity and selectivity results were Cu_0.30_-Mn_0.70_ and Cu_0.40_-Mn_0.60_ catalysts. Their high activity in the studied reaction is explained by their almost identical physicochemical properties which were confirmed by XRD and XPS analysis. The authors also investigated the stability of Cu_0.30_-Mn_0.70_ and commercial CuO/ZnO/Al_2_O_3_ catalysts in combined (oxy–steam) reforming of methanol reaction at 300 °C within 8 h of operation. The only observed difference between the tested systems was the value of the hydrogen selectivity, which was higher for the Cu_0.30_-Mn_0.70_ catalyst. Catalysts operated stably under the reaction conditions, demonstrating constant selectivity towards H_2_ formation and a slight decrease in methanol conversion from 99 to 96% after 8 h of catalyst operation. Other catalysts prepared by Papavasiliou et al. [79] were CuO–CeO_2_ systems prepared by the urea–nitrate combustion method. The activity tests were performed in combined (oxy–steam (CRM) reforming of methanol) reaction. The result presented in Table 4 shows that the catalyst surface area and crystallites size could be controlled and optimized by the preparation method. The authors reported that the optimal fuel to oxidant ratio (urea/nitrates) was 4.17 and the optimal Cu/(Cu + Ce) atomic ratio was 0.15, with the surface area increasing by about 4.3 times, while the methanol conversion increased from 52% to 100%, and the activity increased by about 1.8 times. The comparison of the activity in SRM and CRM showed that under autothermal conditions, the activity of the catalyst was improved. The higher methanol conversion obtained during the CRM process is assigned to better efficiency of the heat transferring in the catalyst bed. In CRM reaction, the methanol conversion obtained at 300 °C was equal to 100% with more than 97% selectivity toward hydrogen formation. However, the CO concentration increased in all cases by increasing the reaction temperature. Liu et al. [85] studied Pd/ZnO catalysts prepared by impregnation and co-precipitation methods and tested their catalytic activity in the oxidative steam-reforming of the methanol process (see Table 4). In addition, the authors also investigated the influence of Pd loading on the activity results in the tested reaction. The Pd/ZnO catalysts with Pd loading below 5% prepared by impregnation method (IP method) showed better activity in the oxidative reforming of methanol process compared to samples prepared by co-precipitation (CP) method. This is due to the higher concentration of the PdZn alloy on the surface of the catalyst prepared by the IP method. Nevertheless, for the higher Pd loading, the Pd/ZnO catalysts prepared by CP method were more active than system prepared by IP method. Based on the obtained results, the authors concluded that the activity of the catalyst strongly depends on the crystal size and the dispersion of the PdZn alloy on the ZnO support. Increasing the size of the Pd crystals on the ZnO support improves the catalytic activity and selectivity of the palladium catalysts. Moreover, the amount of CO generated during the tested process is effectively reduced, along with an increase in the Pd content in the catalytic material.

### 4.2. Effect of the Type of Carrier on the Catalytic Reactivity of the Catalytic Systems Applied in the Oxy–Steam Reforming of Methanol

The type of support and its nature has a great influence on the catalyst material activity and stability [86]. The promotion of copper catalyst by CeO_2_ leads to an increase of the thermal stability in steam-reforming of methanol reaction [78]. CeO_2_ itself can provide mobile oxygen, which has a direct influence on the catalytic activity and may affect the oxidation state of the metal present on the support surface under a reducing environment. Recently, an increase in interest has in the case of catalysts supported on CeO_2_ has been observed. The crystal structure of CeO_2_ is a cubic fluorite network for which it is possible to introduce other cations, such as Si^4+^, Th^4+^, Zr^4+^, Y^3+^, La^3+^, Sc^3+^, Mg^2+^, Ca^2+^ or Cu^2+^ to improve catalytic properties of CeO_2_ [87]. High mobility oxygen present in the CeO_2_ containing systems [88], the strong interaction of CeO_2_ with supported metal—strong metal-support interaction (SMSI) [89], all these features make these systems as promising catalytic materials. The role of promoters of copper supported catalysts is one of the main factors which was brought up in many publications. Promoters have been used to influence the status of copper and improve the activity of the catalyst. Promotion of copper catalyst by CeO_2_ improves activity and stability of the copper-supported catalyst and leads also to the improvement of copper dispersion on catalyst surface and in the same time protects copper crystallites against poisoning and has influence on greatest crystallites formation [90]. Mierczynski et al. [91] studied the influence of copper content in monometallic xCu (where, x = 5, 20, 40 and 60 wt.%) catalysts supported on binary oxide CeO_2_·Al_2_O_3_ on the activity of these systems in the oxy–steam reforming of methanol reaction and the results are presented in Table 4. The authors confirmed that the reactivity of the investigated systems depends on the copper content and its dispersion on the catalyst surface. These studies confirmed that the 20 wt.% of Cu content is the optimum content of copper in order to obtain the highest methanol conversion and reaction rate value compared to other investigated catalytic systems. In addition, the activation energy in the OSRM process for the 20% Cu/CeO_2_·Al_2_O_3_ catalyst was the lowest and equal 66.56 kJ/mol. In other work [23], authors studied both mono-Ni and bimetallic Pd-Ni catalysts supported on CeO_2_, Al_2_O_3_ and CeO_2_·Al_2_O_3_ carriers and they reported about the highest activity of bimetallic 2% Pd-40% Ni/CeO_2_·Al_2_O_3_ system compared to the other investigated catalysts. This catalytic system showed the highest stability and selectivity to H_2_ production in the oxy–steam-reforming of methanol process compared to the systems supported on monoxide. In addition, they investigated the influence of the Ni loading on the catalytic activity of the prepared monometallic systems in the OSRM process. The results showed that system containing 40 wt.% of Ni exhibited the highest methanol conversion value and the highest selectivity to hydrogen formation compared to the rest of the monometallic catalysts. Mierczynski et al. [18] extensively investigated the influence of support composition on the catalytic activity of copper catalysts in order to obtain optimal catalyst composition for the OSRM reaction (see the results presented in Table 4). They prepared various copper catalysts supported on binary oxides systems (ZrO_2_·Al_2_O_3_, where Zr:Al = 2:1, 1:1 and 1:2). The reactivity results performed in the oxy–steam-reforming of methanol confirmed that the highest active system was 20% Cu/ZrO_2_·Al_2_O_3_ (1:2) catalyst. In further studies, the authors compared the physicochemical and catalytic properties of Cu catalyst with Ni system supported on ZrO_2_·Al_2_O_3_ (1:2) carrier. The reactivity measurements showed that the supported copper catalyst was more active than the nickel catalyst. They have found that the catalytic activity of the investigated catalysts strongly dependent on their acidity and sorption properties in relation to methanol. The catalytic tests confirmed the highest activity of copper catalysts supported on ZrO_2_-Al_2_O_3_ (Zr:Al = 0.5) binary oxide promoted by noble metals such as Pd or Rh. The most active systems exhibited the highest specific surface area and the highest number of acidic centers on their surfaces. The XPS measurements showed that the catalyst with the lowest ratio between Cu^0^ and Cu^+^ species present on the catalyst surface exhibited the highest activity in the OSRM process, and the relationship between these species is a critical parameter to achieve highly active systems in the OSRM reaction. Furthermore, they have found that the pre-treatment process of Ni catalysts carried out before the activity tests play a significant role in terms of the catalytic activity of the tested systems in the OSRM process. In other work [22], authors studied the influence of the binary oxide composition of ZnO·Al_2_O_3_ (Zn:Al = 2:1, 1:1, 1:2 and 1:4) on the physicochemical and catalytic properties of nickel supported catalysts. The reactivity tests performed in the oxy–steam-reforming of methanol reaction showed that 20% Ni/ZnO·Al_2_O_3_ (1:1) system was the most active catalysts in the investigated reaction at 300 °C. This result was explained by the easiest reducibility, and the highest acidity of this catalyst compare to other investigated catalysts. Iwasa et al. investigated various metal catalysts Me/ZnO (where Me = Fe, Co, Ni, Pd, Pt, Ir or Ru) catalysts in transformation of methanol in the presence of steam and oxygen [92]. He proved that the best promising system was Pd/ZnO monometallic catalyst. Their high activity was explained by intermetallic Pd-Zn formation. Similar to the Pd/ZnO catalyst, the low selectivity of Pt/ZnO towards CO was assigned to PtZn alloy formation. Whereas, in the case of Ni, Ru, Co and Ir catalyst, no alloy phase was formed after reducing the catalysts at 500 °C. These catalysts showed low methanol conversion and produce a higher concentration of carbon monoxide [93,94]. Palladium is active component for methanol-reforming reactions. It is an effective decomposition catalyst, selectively forming H_2_ and CO when it is supported on metal oxide [95,96,97,98]. Udani et al. [99] also tested Cu-CeO_2_ catalysts in the oxy–steam-reforming of methanol reaction. They investigated copper catalysts with various content of copper. They reported that catalyst with 70 at.% Cu showed the highest catalytic activity in the studied process and was slightly better than commercial SRM catalyst (Synetix 33–5). In addition, the catalytic measurements performed in the OSRM process confirmed the stable operation of the catalytic system at 300 °C. In the literature, data can be found only a few works concerning the use of carbon nanotubes as a carrier of catalytic system used in oxy–steam-reforming of methanol processes. The use of carbon nanotubes (CNTs) or multiwalled carbon nanotubes (MWCNTs) as a carrier for methanol-reforming catalysts is associated with the attractive properties of this material [100]. Carbon nanotubes were applied as a carrier of catalytic materials due to their specific structural morphology and their specific physicochemical properties. In addition, CNTs properties can be modified by functions groups introduced onto their surface. This modification possibilities gives capabilities to obtain specific material. Furthermore, catalysts supported on CNTs can be prepared by different methods such as: impregnation, precipitation, colloidal, electroless plating, and hydrothermal method [100]. The main advantage of carbon nanotubes used as a catalyst carrier is their high purity, high thermal and mechanical stability, the presence of specific interactions on the boundary metal-support, the possibility of adsorption of catalytically active nanoparticles inside or on the external wall of CNTs and their specific electron structure. Good conductivity of carbon nanotubes promotes the “spillover” effect in a place of interfacial boundary created by the active center [101]. Nanoparticles of an active phase dispersed on the functional surface of the support (CNTs) make that created active centers are easily accessible to the reactants. All remaining above properties of CNTs can directly affect the catalytic activity and selectivity [102,103,104,105,106]. All mentioned properties of CNTs material have an important influence on the catalytic activity and selectivity of the synthesized catalysts. Mierczynski et al. [19] reported the potential usages of MWCNTs in the OSRM reaction to hydrogen generation. They have confirmed that magnetite, metallic iron and defects formed by encapsulated or removal of metal particles play an important role in the oxy–steam-reforming of methanol. Their results indicate that MWCNTs are potential carriers for various metallic catalysts which can be applied in methanol processing reactions.

### 4.3. Role of Promotors Addition on the Catalytic and Physicochemical Properties of Catalytic Materials Tested in the Oxy–Steam Reforming of Methanol Reaction

**Figure 7:**
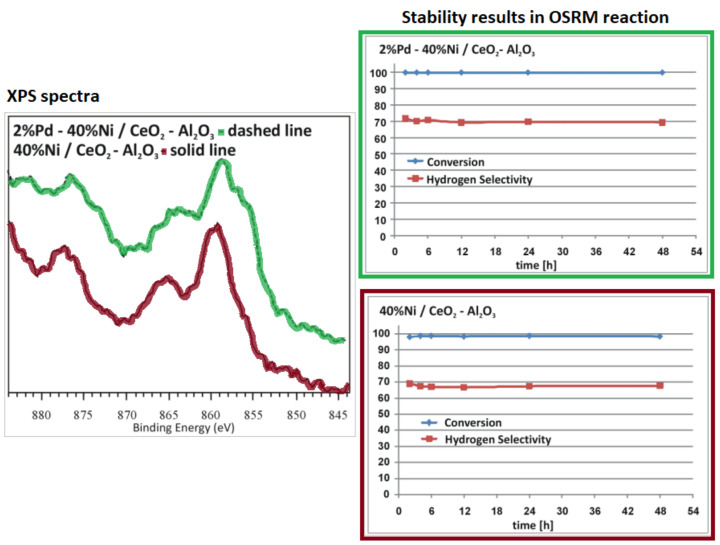
XPS and stability results during 48 h of the OSRM reaction performed at 250 °C after reduction 1 h at 300 °C [23].

**Figure 8:**
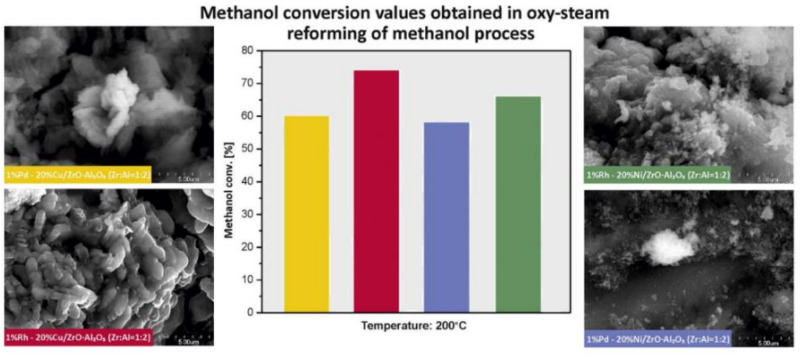
The activity results of Rh(Pd)-Cu(Ni)/ZrO_2_·Al_2_O_3_ (1:2) catalysts in the OSRM process [18].

The role of promoters of copper supported catalysts is one of the main factors which was brought up in many publications. Promoters have been used to influence the status of copper and improve the activity of the catalyst. Modification of copper catalyst by CeO_2_ improves activity, the addition of ZnO and ZrO_2_ cause increase of catalyst surface, and also stabilize crystallites size of copper, and in the same time protects crystallites against their aggregations. Additionally, ZrO_2_ stabilizes the copper Cu^+^ ions on the catalyst surface [39]. Agrell et al. [107] determined the influence of ZrO_2_ and Al_2_O_3_ promotion on physicochemical and catalytic properties of Cu/ZnO catalyst (see the results presented in Table 4). The authors observed an increase in copper dispersion after adding ZrO_2_ to Cu/ZnO catalyst. While the structural promoter Al_2_O_3_ provides a larger surface into catalytic systems, which directly leads to high dispersion of copper [30,107]. The authors observed an increase in both the total surface area and the degree of copper dispersion after the introduction of Al_2_O_3_ into the catalytic system. They also performed the activity tests of catalytic systems in the steam, partial oxidation and oxy–steam reforming of methanol processes. They reported that ZrO_2_ containing catalysts were more active compared to other investigated catalysts. Cu/ZnO/ZrO_2_ and Cu/ZnO/ZrO_2_/A1_2_O_3_ catalysts exhibited high activity in oxy–steam-reforming of methanol. It should be emphasized that the Cu/ZnO catalyst was more efficient at low CH_3_OH conversion values. In the case of this system, the authors observed a lower amount of CO formed during the studied process compared to the amount of carbon monoxide generated during the steam-reforming reaction of methanol (SRM). As part of the work, the authors conducted stability studies in the process of oxy–steam reforming of methanol on Cu/ZnO and Cu/ZnO/ZrO_2_/Al_2_O_3_ catalysts at a temperature of 260 °C within 20 h of operation. The results showed that ZrO_2_ addition improves the lifetime of the studied catalyst. A similar promotional effect attributed to Cr_2_O_3_ was observed in other works [40,108]. The addition of this promotor acts as a stabilizer of the copper structure, protecting it against sintering. The promotion effect of palladium on the catalytic activity and selectivity towards hydrogen production of nickel-supported catalysts in the OSRM was studied by Mierczynski et al. [23]. The obtained results showed that the palladium addition facilitates the reducibility of the nickel supported catalysts. In addition, they confirmed the highest activity of Pd-Ni catalyst in the studied process, which exhibited the highest selectivity towards hydrogen production and towards carbon monoxide formation in the investigated reaction. Liu et al. [109] investigated the effects of modifier (Zr, Fe, Mg, Al and Cr) of a Pd/ZnO catalyst prepared by co-precipitation method on the catalytic activity in the oxidative reforming of methanol reaction. The addition of modifier to Pd/ZnO catalyst in the form of metal significantly modifies its physicochemical and catalytic properties in an oxidative reforming of methanol process. The ZnO crystal size decreases as a result of modifier addition. During high-temperature reduction treatment, the smaller PdZn alloy particles are formed. The introduced modifier can interact with ZnO, which leads to the formation of binary oxides systems such as ZnAl_2_O_4_, ZnCr_2_O_4_ and ZnFe_2_O_4_. The promotion effect of a small amount of Cr or Fe into Pd/ZnO catalyst results in decreases in the amount of CO formed during the process and increased the selectivity toward H_2_ production. However, the CO selectivity of the Pd/ZnO catalyst increases as a result of Mg, Zr or Al addition. Mierczynski et al. [18,22,23] also investigated the influence of Pd addition into Ni catalysts on their activity results in the OSRM process. The authors confirmed that the palladium addition has a promotion effect on the performance of Ni supported catalyst in the OSRM reaction. They proved that Pd facilitates the NiO reduction by the spillover effect occurring between Pd and NiO species. This phenomenon is related to the generation of new adsorption centers on the catalyst surface, which may be responsible for increasing the activity and selectivity of the bimetallic catalyst towards H_2_ formation. They also reported that the activity in the OSRM reaction strongly depends on the Ni content in the investigated system and their reduced properties. The acidity measurement also confirmed that acid sites play a crucial role in the oxy–steam-reforming of the methanol process. In addition, the presence of acid centers located close to high dispersed metal centers may have a significant influence on the reactivity of the tested catalyst systems. Catalytic activity tests performed for 48 h confirmed the stable operation of the Pd-Ni bimetallic catalyst. (see Figure 7). In another work [18], the authors also reported the high activity of bimetallic Rh-Cu, Rh-Ni and Pd-Cu, Pd-Ni catalysts tested in the OSRM process. They confirmed that the most active system in the studied process was 0.5% Rh-20% Cu/ZrO_2_·Al_2_O_3_ (1:2) catalyst (see Figure 8). They also claimed that the occurrence of Cu^0^ and Cu^+^ species and their ratio is a critical parameter to achieve highly active systems in the OSRM reaction.

**Figure 9:**
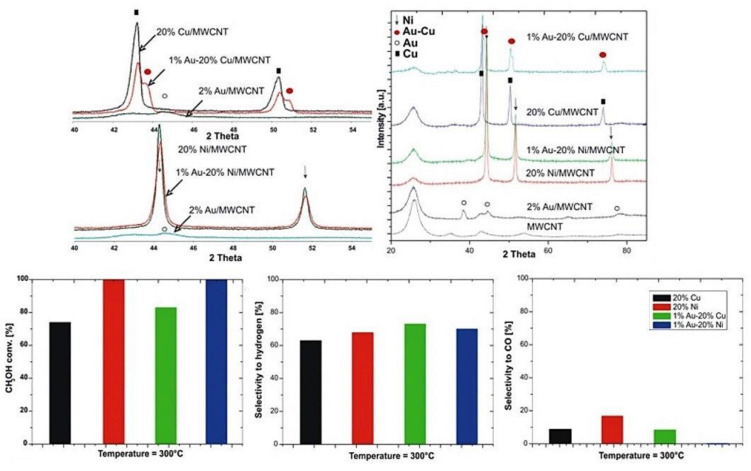
Phase composition studies of spent catalysts and reactivity results of bimetallic Au–Cu and Au–Ni system in the OSRM process [17].

In other work [92], the authors reported the potential capability to use palladium as a promoter for methanol-reforming catalysts. Despite the relatively high price of gold, even a small addition of gold can significantly improves the activity of bimetallic catalysts through the formation of intermetallic compound Au–Cu, Au–Ni, which catalyze the OSRM process. Since Haruta’s discovery that catalysts containing nanosized gold particles have extraordinary activities for reactions, including CO oxidation [110], there has been substantial interest in their use and the origin of their exceptional catalytic properties. The use of gold as a promoter of the active phase (Cu, Ni) results in lowering the reaction temperature and improves selectivity toward hydrogen and carbon dioxide formation. The selectivity improvement is associated with the surface modification of copper and nickel by gold atoms. These new atoms can generate new adsorption centers, which may be involved in the dissociative adsorption of methanol or adsorption of atomic oxygen as a result of dissociation of previously adsorbed methanol. Adsorbed oxygen atoms can leave the surface of the catalyst as a product of CO_2,_ thereby reducing the formation of CO. Mierczynski et al. [17,111,112] for the first time investigated the physicochemical and catalytic activity of the mono-(Cu, Ni) and bimetallic (Au–Cu, Au–Ni) catalysts supported on multiwalled carbon nanotubes (MWCNTs) in oxy–steam-reforming of methanol process (see Figure 9). They proved that the AuCu and AuNi alloys are formed in the case of bimetallic Au–Cu/MWCNTs and Au–Ni/MWCNTs catalyst after their reduction. These results were confirmed by XRD, XPS and SEM-EDS measurements. The spillover effect between metallic gold and nickel (copper) oxide was confirmed by TPR-H_2_ studies. The bimetallic Au–Cu catalyst exhibited a significant improvement of the activity and selectivity towards hydrogen formation compared to the monometallic systems tested in the OSRM process at higher temperatures (300 °C). The higher activity and selectivity towards H_2_ production is explained by an alloy AuCu formation. The Au–Ni/MWCNTs catalyst showed the lowest selectivity towards CO formation at 200 and 300 °C, which is very important from the application point of view of these catalytic systems in fuel cell technology.

### 4.4. Role of Active Phase Composition on Reactivity Properties of Catalytic Materials Applied in Oxy–Steam Reforming of Methanol

The type of an active phase has an important influence on the reactivity properties of synthesized catalysts in methanol processing reactions. It is well known that copper and nickel catalysts supported on metal oxide support are highly active in methanol processing reactions, as evidenced by a number of papers published in reputable journals concerning copper and nickel catalysts using in methanol reforming processes. In addition, transition metal catalysts and noble metals are also used in oxy–steam reforming of methanol reaction. The activity results obtained in steam-reforming of methanol reaction on Pd/ZnO catalyst showed that palladium is an effective catalyst in the studied process. Pd/ZnO system showed high selectivity for CO_2_ production in the SRM process. The high selectivity towards CO_2_ formation is explained by the Pd-Zn alloys formed on the catalyst surface [113]. These palladium alloys are formed at moderate temperatures under reducing conditions [114,115]. Iwasa et al. [92] also compared the catalytic properties of Pd/ZnO catalyst with copper-containing systems (Cu/ZnO, Cu/ZrO_2_ and Cu/SiO_2_) in steam reforming of methanol reaction. The methanol conversion and hydrogen concentration obtained for Pd/ZnO were higher compared to Cu/ZrO_2_ and Cu/SiO_2_ catalysts and slightly lower than for Cu/ZnO system. The influence of Pd loading on Pd/ZnO catalyst was examined, and obtained results showed the CH_3_OH conversion increased with increasing Pd loading. In the presence of oxygen, the distribution of reaction products depends on Pd loading. The increase od Pd concentration in Pd/ZnO catalyst results in increasing of H_2_ and decreasing of CO level in an obtained reaction product. The 1% Pd/ZnO catalyst exhibited the highest concentration of H_2_O in the product in contrast to the 10% Pd/ZnO catalyst for which water was not a product of the reaction. Xu et al. [116] have been investigated Pt-based alloys, including Pt–Cr, Pt–Fe, Pt–Co, Pt–Ni and Pt–Au, as methanol-tolerant cathode catalysts. They reported that the Pt–Au catalyst had been the most promising one in terms of both the catalytic activity and stability. Studies indicate that the methanol-tolerant mechanism of the Pt-based alloys can be attributed to the diluted Pt sites for methanol dehydrogenation as compared with the pure Pt catalyst.

**Figure 10:**
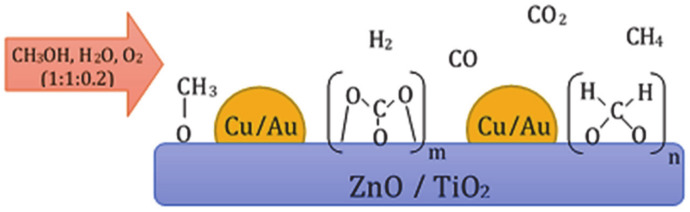
The species formed during the combined reforming of methanol process [117].

Manzoli et al. [117] investigated CuO/ZnO, Au/ZnO, Cu/TiO_2_ and Au/TiO_2_ catalysts in the decomposition and oxy–steam reforming of methanol process. The catalyst systems supported on ZnO were prepared by the co-precipitation method, while catalysts containing TiO_2_ oxide were prepared by deposition–precipitation method. The catalytic activity of the investigated systems expressed as H_2_/CO ratio can be described by the following row: Cu/ZnO > Cu/TiO_2_ > Au/ZnO > Au/TiO_2_, respectively. In contrast, the CO_2_/CO ratios formed during the investigated process for Au containing systems were higher than for Cu catalysts. Authors indicated that copper catalysts used in the OSRM process (reaction mixture—CH_3_OH-H_2_O-O_2_ in a molar ratio equal 1:1:0.2) at 200 °C exhibited a higher molar ratio of H_2_/CO compared to the process realized over gold-containing systems. However, the produced CO_2_/CO ratio in the final product obtained in the OSRM process over gold catalysts was higher than in the case of copper catalysts, which means that the use of a gold catalyst reduces the amount of CO generated in the reaction. In addition, the authors reported about lower activity of the TiO_2_ supported catalysts in the OSRM process. These results are related to the high selectivity of TiO_2_-containing systems towards methane formation, which reduces the amount of produced hydrogen. Figure 10 present the surface and gaseous species which are produced during the OSRM process. They have reported about undefined C-containing species (possibly polyoxymethylene or bidentate carbonate) formed during contact of the reaction mixture with the catalyst surface.

**Figure 11:**
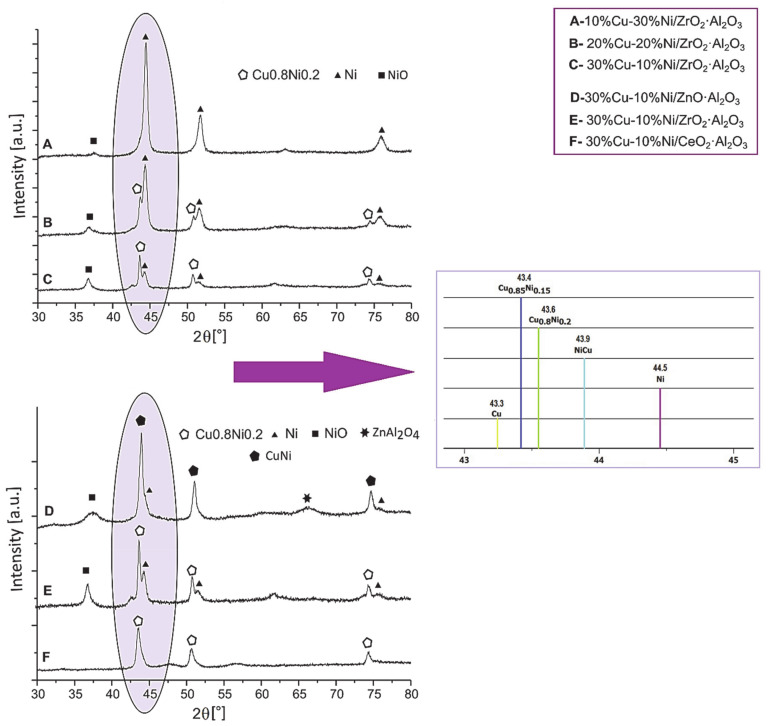
XRD patterns of spent bimetallic Cu–Ni systems [4].

Literature data show that bimetallic catalysts Ni–Cu have also been extensively studied in the oxy–steam reforming of both ethanol [118] and methanol reactions [119]. Catalytic tests of Ni_x_Cu_y_-Al catalysts with different Ni to Cu contents [120] carried out in the studied processes showed that the Ni-Cu alloy containing catalyst had better performance in reforming of ethanol and methanol process compared to monometallic copper catalysts. It has also been shown that the introduction of Cu into nickel-based catalysts prevents carbon deposition and sintering of the active phase of catalysts used in the methanol-reforming process. The addition of Cu to the nickel catalyst also prevents the formation of methane and increases the stability of the Ni catalyst during the methanol-reforming process [119]. While, the addition of nickel into copper catalysts also improves Cu dispersion compared to the dispersion of copper species observed in the case of the Cu/ZnO/Al_2_O_3_ catalyst. The authors reported that a bimetallic 5% Ni-5% Cu/Al_2_O_3_ catalyst was very active in both methane and methanol steam reforming reactions compared to commercial catalysts. Perez-Hernandez et al. [121] also studied Cu/ZrO_2_, Ni/ZrO_2_ and Cu–Ni/ZrO_2_ catalysts in oxidative steam reforming of methanol reaction in order to produce H_2_-rich gas at relatively low temperature (see the results presented in Table 4). The activity results showed that the monometallic Ni/ZrO_2_ catalyst was more active than the Cu/ZrO_2_ system in the OSRM reaction at higher temperatures. However, the bimetallic Cu–Ni/ZrO_2_ catalyst showed the best catalytic performance at low reaction temperatures compared to the monometallic catalysts. This activity result was attributed to the bimetallic nanoparticles present on the catalyst surface with different Cu/Ni weight ratios. The investigated catalysts showed a similar selectivity toward H_2_ production equal about 60–70% at higher reaction temperatures. The Cu–Ni/ZrO_2_ system exhibited high selectivity toward CO formation, which is related to the presence of bimetallic nanoparticles on the catalyst surface. Mosinska et al. [4] also studied the bimetallic x Cu-y Ni (where x(y) = 10, 20 and 30 wt.%) catalysts supported on binary oxides (ZnO·Al_2_O_3_, CeO_2_·Al_2_O_3_, ZrO_2_·Al_2_O_3_), and the results are given in Table 4. Authors reported that the hydrogen can be effectively produce in the OSRM process over an investigated Cu–Ni catalyst systems. The 30% Cu–10% Ni/ZrO_2_·Al_2_O_3_ system was the most active system at 160 °C. The high reactivity of this catalyst is related with the Cu_0.8_Ni_0.2_ alloy formation which was confirmed by XRD, ToF-SIMS and XPS techniques (see Figure 11).

In addition, the most active system in the studied process at 200 °C was 30% Cu–10% Ni/CeO_2_·Al_2_O_3_ catalyst. This catalytic system was easily reducible and showed the presence of the Cu0.8Ni0.2 alloy on its surface. The authors also confirmed that the alloy composition is an important parameter influencing the reactivity of the bimetallic Cu–Ni catalysts in the OSRM process. At the same time, the bimetallic 30% Cu–10% Ni/ZnO·Al_2_O_3_ catalyst containing the equimolar Cu–Ni alloy and irreducible support showed the lowest activity in the studied oxy–steam-reforming of methanol reaction. The authors of the work [122] investigated the catalytic activity of the Au/CeO_2_-0.135 system in the OSRM process. The results showed that it has the highest methanol conversion and the H_2_ production rate for oxidative steam reforming of methanol (OSRM). The reducibility and gold particle size played a crucial role in determining the H_2_/CO_2_ ratio at the temperature of 200 °C. The authors reported that the reducibility of the catalytic material, small gold particle size and cationing gold centers as active sites play a crucial role in the catalytic activity of prepared catalysts in the OSRM process. Wang et al. [123] reported the high activity of the ZnO–Cr_2_O_3_/CeO_2_–ZrO_2_ catalyst in oxidative steam reforming of methanol at higher temperatures, which exhibited fast reaction rates compared to conventional Cu-based catalyst. The investigated catalysts produced much lower CO compared to the Cu catalyst and can be used to determine detailed kinetic modeling and design of optimal parameters of the OSRM reactors.

## 5. Conclusions

This work provides valuable information about catalyst systems used in reforming methanol processes. This paper sheds light on the role of the catalyst in oxy–steam reforming of methanol reaction and presents possible methods of modification of catalytic systems in order to achieve high active, stable and selective catalysts of this process. It was shown that the selection of the suitable preparation method, type of support, the addition of promoters to copper, nickel or transition metal catalysts have a great influence on the catalyst performance in the OSRM reaction. The mechanism of the OSRM process on the copper-based catalyst surface was also discussed. However, it is still a controversial issue to show the clear approach of the OSRM mechanism. In this review, we presented the latest data concerning the catalytic materials and mechanism approach applied in the oxy–steam reforming of methanol process. The presented data may become the basis for the development of the industrial catalyst used in the methanol processing reaction and may also contribute to the development of new technologies based on fuel cells.

## Figures and Tables

**Table 1:** The coefficients of combination for two models of autothermal reforming of methanol process (ATRM) [24].

		POM-SRM	TOM-SRM
**SRM**	CH_3_OH + H_2_O → CO_2_ + 3H_2_	3	$3\frac{2}{3}$
**POM**	CH_3_OH + $\frac{1}{2}$O_2_ → CO_2_ + 2H_2_	1	-
**TOM**	CH_3_OH + $\frac{3}{2}$O_2_ → CO_2_ + 2H_2_O	-	$\frac{1}{3}$
∑ATRM	4CH_3_OH + 3H_2_O + $\frac{1}{2}$O_2_ + 2N_2_ → 4CO_2_ + 11H_2_ + 2N_2_		

**Table 2:** Activation energy for the oxy–steam-reforming of methanol process [49].

Catalyst	E_a_ (cal mol^−1^)
Cu(5)Zn(50)Al(45)	24
Cu(15)Zn(48)Al(37)	27
Cu(18)Zn(33)Al(49)	16
Cu(45)Zn(31)Al(24)	22
Cu(75)Zn(25)Al(0)	-

**Table 3:** Reaction conditions and catalytic materials applied in the studied process.

References	Investigated Mechanism	Employed Catalyst	Operating Conditions				
			Total Flow [cm^3^/min]	Temperature [K]	Pressure [atm]	H_2_O/CH_3_OH	O_2_/CH_3_OH
[56]	Selective oxidation of CH_3_OH to H_2_CO	Copper (110)	-	295	-	-	-
[59]	SRM	Copper containing catalyst	-	360–573	1	1.5	-
[60]	SRM	Cu/ZnO/Al_2_O_3_	-	433–473	1	-	-
[61]	SRM	CuO/ZnO/ZrO_2_/Al_2_O_3_	-	473 and 573	1	1.3	-
[62]	SRM	Cu/ZnO/Al_2_O_3_	230	448–623	1	1.3	-
[77]	SRM	Cu/ZnO/Al_2_O_3_	50	433–533	1–35	0–1.2	-
[45]	SRM	Cu/ZnO/Al_2_O_3_	50	433–533	1–35	0–1.2	-
[63]	SRM	Cu/ZrO_2_/CeO_2_	-	523	1	1.0	-
[66]	SRM	Cu/ZnO/Al_2_O_3_	-	443–533	1	-	-
[67]	SRM	Cu/Zn/Zr/Al	38.6	413–618	1	1.3	-
[47]	SRM	copper–silica	-	433 and 453	1	-	-
[69]	SRM	copper–silica aerogel	-	423–673	1	2.0	-
[70]	OSRM	Cu/ZnO/Al_2_O_3_	120	473–633	1	1.1	0.3
[71]	POM	Cu/ZnO/Al_2_O_3_	-	453 and 493	-	-	0.1–0.5
[72]	OSRM	Cu/ZrO_2_/CeO_2_/Al_2_O_3_	-	473–573	1	1.5	0.1–0.2
[73]	OSRM	Cu/CeO_2_/Al_2_O_3_	-	473–573	1	1.5	0–0.5
[74]	OSRM	Cu/ZnO/Al_2_O_3_ Cu/ZnO/Al_2_O_3_/Cr_2_O_3_	100	538–548	1.28	1	0.9
[48]	OSRM	Cu/ZnO/Al_2_O_3_	-	473–673	-	1.1	0.12

**Table 4:** The physicochemical and catalytic characterization of the various catalysts applied in OSR of methanol reaction.

Catalyst	Preparation Method	S_BET_ (m^2^/g)	Metal Dispersion (%)	Reduction Temp. (°C)	Reduction Time (h)	$\frac{H_{2}O}{\mathrm{methanol}}$	$\frac{O_{2}}{\mathrm{methanol}}$	W/F (gscm^−3^)	GHSV (h^−1^)	T_OSR_ (°C)	Catalyst Weight (g)	CH_3_OH Conv. (%)	H_2_	CO	CO_2_
CuO/ZnO/Al_2_O_3_ [42]	CP	94	-	350–450	2	1.43	0.47	-	-	230	0.3	100.0	71.0 ^y^	0.1 ^y^	28.9 ^y^
Cu_0.30_Mn_0.70_ [39]	UNC	8	-	320	2	1.26	0.10	0.257	-	240	0.3	100.0	97.0 ^s^	3.0 ^s^	-
CuO–CeO_2_ (mol.rat. = 0.15, u/n rat. = 1.2) [79]	UNC	10	-	-	-	1.50	0.10	0.257	-	300	0.3	56.0	90.3 ^s^	2.1 ^s^	-
CuO–CeO_2_ (mol.rat. = 0.15, u/n rat. = 1.2) [79]	UNC	10	-	-	-	1.50	0.10	0.257	-	340	0.3	5.7	75.0 ^s^	0.8 ^s^	-
CuO–CeO_2_ (mol.rat. = 0.15, u/n rat. = 2.38) [79]	UNC	5	-	-	-	1.50	0.10	0.257	-	300	0.3	45.0	84.3 ^s^	4.8 ^s^	-
CuO–CeO_2_ (mol.rat. = 0.15, u/n rat. = 2.38) [79]	UNC	5	-	-	-	1.50	0.10	0.257	-	240	0.3	12.0	69.0 ^s^	3.1 ^s^	-
CuO–CeO_2_ (mol.rat. = 0.15, u/n rat. = 3.30) [79]	UNC	20	-	-	-	1.50	0.10	0.257	-	300	0.3	95.0	96.6 ^s^	3.4 ^s^	-
CuO–CeO_2_ (mol.rat. = 0.15, u/n rat. = 3.30) [79]	UNC	20	-	-	-	1.50	0.10	0.257	-	240	0.3	32.4	92.6 ^s^	1.0 ^s^	-
CuO–CeO_2_ (mol.rat. = 0.15, u/n rat. = 4.17) [79]	UNC	43	-	-	-	1.50	0.10	0.257	-	300	0.3	100	95.9 ^s^	4.0 ^s^	-
CuO–CeO_2_ (mol.rat. = 0.15, u/n rat. = 4.17) [79]	UNC	43	-	-	-	1.50	0.10	0.257	-	240	0.3	36.5	93.7 ^s^	0.8 ^s^	-
Cu(5)/CeO_2_·Al_2_O_3_ [91]	IP	126	2.14 *	300	1	-	-	-	26,700	200	0.2	97.7	69.1 ^s^	0 ^s^	30.6 ^s^
Cu(20)/CeO_2_·Al_2_O_3_ [91]	IP	101	0.16 *	300	1	-	-	-	26,700	200	0.2	99.6	68.2 ^s^	0 ^s^	31.7 ^s^
Cu(40)/CeO_2_·Al_2_O_3_ [91]	IP	90	0.13 *	300	1	-	-	-	26,700	200	0.2	87.9	65.7 ^s^	0 ^s^	33.8 ^s^
Cu(60)/CeO_2_·Al_2_O_3_ [91]	IP	30	-	300	1	-	-	-	26,700	200	0.2	9.6	28.5 ^s^	0 ^s^	71.5 ^s^
Ni(5)/CeO_2_·Al_2_O_3_ [23]	IP	132	1.06 *	300	1	-	-	-	26,700	250	0.2	2	30.5 ^s^	-	25 ^s^
Ni(20)/CeO_2_·Al_2_O_3_ [23]	IP	128	1.16 *	300	1	-	-	-	26,700	250	0.2	31	33 ^s^	-	55.6 ^s^
Ni(40)/CeO_2_·Al_2_O_3_ [23]	IP	78	0.56 *	300	1	-	-	-	26,700	250	0.2	98	68.9 ^s^	13.3 ^s^	14.3 ^s^
Ni(60)/CeO_2_·Al_2_O_3_ [23]	IP	133	0.69 *	300	1	-	-	-	26,700	250	0.2	77	58.2 ^s^	27.7 ^s^	14.1 ^s^
Ni(40)/CeO_2_ [23]	IP	34	0.63 *	300	1	-	-	-	26,700	250	0.2	85	68.6 ^s^	22.6 ^s^	0.5 ^s^
Ni(40)/Al_2_O_3_ [23]	IP	58	0.28 *	300	1	-	-	-	26,700	250	0.2	20.5	53.4 ^s^	-	25.2 ^s^
Pd(2)–Ni(40)/CeO_2_·Al_2_O_3_ [23]	SIP	42	-	300	1	-	-	-	26,700	250	0.2	99.9	71.5 ^s^	14.9 ^s^	9.1 ^s^
Pd/ZnO [85]	IP	-	-	400	2	1.50	0.10	-	110,000	250	0.3	-	-	-	-
Pd/ZnO [85]	CP	-	-	400	2	1.50	0.10	-	110,000	250	0.3	-	-	-	-
Cu/ZnO [107]	CP	49	9.6 *	250–300	1	1.30	0.20	-	-	300	0.5	90.0	50.0 ^m^	0.07 ^m^	20.0 ^m^
Cu/ZnO/Al_2_O_3_ [107]	CP	92	11.3 *	250–300	1	1.30	0.20	-	-	325	0.5	90.0	-	0.13 ^m^	-
Cu/ZnO/ZrO_2_ [107]	CP	82	13.2 *	250–300	1	1.30	0.20	-	-	295	0.5	90.0	-	0.04 ^m^	-
Cu/ZnO/ZrO_2_/Al_2_O_3_ [107]	CP	116	23.2 *	250–300	1	1.30	0.20	-	-	295	0.5	90.0	-	0.05 ^m^	-
Cu(20)/ZrO_2_·Al_2_O_3_ (2:1) [18]	IP	143	-	300	1	-	-	-	26,700	200	0.2	22	41 ^s^	0^s^	59 ^s^
Cu(20)/ZrO_2_·Al_2_O_3_ (1:1) [18]	IP	138	-	300	1	-	-	-	26,700	200	0.2	46	70 ^s^	0 ^s^	29 ^s^
Cu(20)/ZrO_2_·Al_2_O_3_ (1:2) [18]	IP	167	-	300	1	-	-	-	26,700	200	0.2	58	68 ^s^	0 ^s^	31 ^s^
Ni(20)/ZrO_2_·Al_2_O_3_ (1:2) [18]	IP	116	-	300	1	-	-	-	26,700	300	0.2	94	70 ^s^	25 ^s^	5 ^s^
Ni(20)/ZrO_2_·Al_2_O_3_ (1:2) [18]	IP	116	-	500	1	-	-	-	26,700	300	0.2	61	65 ^s^	0 ^s^	22 ^s^
Pd(1)–Cu(20)/ ZrO_2_·Al_2_O_3_ (1:2) [18]	SIP	171	-	300	1	-	-	-	26,700	200	0.2	60	66 ^s^	0 ^s^	33 ^s^
Rh(0.5)–Cu(20)/ ZrO_2_·Al_2_O_3_ (1:2) [18]	SIP	-	-	300	1	-	-	-	26,700	200	0.2	86	68 ^s^	14 ^s^	18^s^
Rh(1)–Cu(20)/ ZrO_2_·Al_2_O_3_ (1:2) [18]	SIP	164	-	300	1	-	-	-	26,700	200	0.2	74	71 ^s^	4 ^s^	25 ^s^
Rh(2)–Cu(20)/ ZrO_2_·Al_2_O_3_ (1:2) [18]	SIP	-	-	300	1	-	-	-	26,700	200	0.2	61	60 ^s^	18 ^s^	22 ^s^
Pd(1)–Ni(20)/ ZrO_2_·Al_2_O_3_ (1:2) [18]	SIP	120	-	300	1	-	-	-	26,700	200	0.2	58	63 ^s^	19^s^	18 ^s^
Rh(1)–Ni(20)/ ZrO_2_·Al_2_O_3_ (1:2) [18]	SIP	123	-	300	1	-	-	-	26,700	200	0.2	66	64 ^s^	18^s^	18 ^s^
Ni(20)/ZnO·Al_2_O_3_ (2:1) [22]	IP	108	-	300	1	-	-	-	26,700	300	0.2	78	76 ^s^	0 ^s^	24 ^s^
Ni(20)/ZnO·Al_2_O_3_ (1:1) [22]	IP	123	-	300	1	-	-	-	26,700	300	0.2	99	76 ^s^	0 ^s^	24 ^s^
Ni(20)/ZnO·Al_2_O_3_ (1:2) [22]	IP	231	-	300	1	-	-	-	26,700	300	0.2	73	40 ^s^	10 ^s^	24 ^s^
Ni(20)/ZnO·Al_2_O_3_ (1:4) [22]	IP	246	-	300	1	-	-	-	26,700	300	0.2	83	65 ^s^	0 ^s^	21 ^s^
Pd(0.5)–Ni(20)/ ZnO·Al_2_O_3_ (1:1) [22]	SIP	106	-	300	1	-	-	-	26,700	300	0.2	99	73 ^s^	10 ^s^	17 ^s^
Pd(2)–Ni(20)/ ZnO·Al_2_O_3_ (1:1) [22]	SIP	104	-	300	1	-	-	-	26,700	300	0.2	99	72 ^s^	8 ^s^	20 ^s^
Pd(6.5)/ZnO [109]	CP	-	-	400/500	2	1.50	0.10	-	-	250	0.3	-	-	-	-
Pd(6.5)/ZnO–ZrO_2_ [109]	CP	-	-	400/500	2	1.50	0.10	-	-	250	0.3	-	-	-	-
Pd(6.5)/ZnO–Fe_3_O_4_ [109]	CP	-	-	400/500	2	1.50	0.10	-	-	250	0.3	-	-	-	-
Pd(6.5)/ZnO–MgO [109]	CP	-	-	400/500	2	1.50	0.10	-	-	250	0.3	-	-	-	-
Pd/(6.5)ZnO–Cr_2_O_3_ [109]	CP	-	-	400/500	2	1.50	0.10	-	-	250	0.3	-	-	-	-
Pd/(6.5)ZnO–Al_2_O_3_ [109]	CP	-	-	400/500	2	1.50	0.10	-	-	250	0.3	-	-	-	-
Pd(10)/ZnO [92]	CP	-	-	0–500	1	-	-	-	-	300	0.1	100	67 ^m^	6 ^m^	27 ^m^
Pt(10)/ZnO [92]	CP	-	-	0–500	1	-	-	-	-	300	0.1	100	70 ^m^	2 ^m^	28 ^m^
Co(10)/ZnO [92]	CP	-	-	0–500	1	-	-	-	-	300	0.1	53	43 ^m^	5 ^m^	30 ^m^
Ni(10)/ZnO [92]	CP	-	-	0–500	1	-	-	-	-	300	0.1	96	54 ^m^	23 ^m^	13 ^m^
Ir(10)/ZnO [92]	IP	-	-	0–500	1	-	-	-	-	300	0.1	59	49 ^m^	3 ^m^	31 ^m^
Ru(10)/ZnO [92]	CP	-	-	0–500	1	-	-	-	-	300	0.1	88	48 ^m^	25 ^m^	11 ^m^
Pd(10)/ZnO [92]	CP	-	-	0–500	1	-	-	-	-	220	0.1	89	60 ^m^	3 ^m^	27 ^m^
Pd(10)/SiO_2_ [92]	CP	-	-	0–500	1	-	-	-	-	220	0.1	22	2 ^m^	10 ^m^	21 ^m^
Pd(1)/CeO_2_ [92]	CP	-	-	0–500	1	-	-	-	-	220	0.1	39	11 ^m^	14 ^m^	21 ^m^
Pd(1)/ZnO [92]	CP	-	-	0–500	1	-	-	-	-	220	0.1	55	35 ^m^	14 ^m^	24 ^m^
Pd(5)/ZnO [92]	CP	-	-	0–500	1	-	-	-	-	220	0.1	80	60 ^m^	10 ^m^	25 ^m^
Cu(25)/ZnO [92]	CP	-	-	0–500	1	-	-	-	-	220	0.1	99	20 ^c^	-	-
Cu(25)/ZrO_2_ [92]	IP	-	-	0–500	1	-	-	-	-	220	0.1	75	13 ^c^	-	-
Cu(25)/SiO_2_ [92]	IP	-	-	0–500	1	-	-	-	-	220	0.1	5	0.1 ^c^	-	-
Cu/ZrO_2_ [121]	DP	33	-	25–300	1	-	-	-	30,000	310	0.1	40	68 ^m^	2 ^m^	98 ^m^
Cu/ZrO_2_ [121]	DP	33	-	25–300	1	-	-	-	30,000	350	0.1	50	70 ^m^	10 ^m^	90 ^m^
Ni/ZrO_2_ [121]	DP	34	-	25–300	1	-	-	-	30,000	310	0.1	30	60 ^m^	19 ^m^	80 ^m^
Ni/ZrO_2_ [121]	DP	34	-	25–300	1	-	-	-	30,000	350	0.1	100	62 ^m^	80 ^m^	15 ^m^
Cu–Ni/ZrO_2_ [121]	DP	35	-	25–300	1	-	-	-	30,000	310	0.1	90	72 ^m^	87 ^m^	13 ^m^
Cu–Ni/ZrO_2_ [121]	DP	35	-	25–300	1	-	-	-	30,000	350	0.1	99	63 ^m^	80 ^m^	20 ^m^
Cu(10)–Ni(30)/ ZrO_2_·Al_2_O_3_ [4]	CIP	120	-	300	1	-	-	-	26,700	160	0.2	22	3.0 ^y^	0 ^s^	100 ^s^
Cu(10)–Ni(30)/ ZrO_2_·Al_2_O_3_ [4]	CIP	120	-	300	1	-	-	-	26,700	200	0.2	85	2.2 ^y^	48 ^s^	52 ^s^
Cu(20)–Ni(20)/ ZrO_2_·Al_2_O_3_ [4]	CIP	142	-	300	1	-	-	-	26,700	160	0.2	35	3.0 ^y^	0 ^s^	100 ^s^
Cu(20)–Ni(20)/ ZrO_2_·Al_2_O_3_ [4]	CIP	142	-	300	1	-	-	-	26,700	200	0.2	86	2.0 ^y^	48 ^s^	52 ^s^
Cu(30)–Ni(10)/ ZrO_2_·Al_2_O_3_ [4]	CIP	119	-	300	1	-	-	-	26,700	160	0.2	79	3.0 ^y^	0 ^s^	100 ^s^
Cu(30)–Ni(10)/ ZrO_2_·Al_2_O_3_ [4]	CIP	119	-	300	1	-	-	-	26,700	200	0.2	91	2.3 ^y^	39 ^s^	61 ^s^
Cu(30)–Ni(10)/ CeO_2_·Al_2_O_3_ [4]	CIP	120	-	300	1	-	-	-	26,700	160	0.2	26	3.0 ^y^	0 ^s^	100 ^s^
Cu(30)–Ni(10)/ CeO_2_·Al_2_O_3_ [4]	CIP	120	-	300	1	-	-	-	26,700	200	0.2	96	2.1 ^y^	30 ^s^	70 ^s^
Cu(30)–Ni(10)/ ZnO·Al_2_O_3_ [4]	CIP	150	-	300	1	-	-	-	26,700	160	0.2	19	3.0 ^y^	0 ^s^	100 ^s^
Cu(30)–Ni(10)/ ZnO·Al_2_O_3_ [4]	CIP	150	-	300	1	-	-	-	26,700	200	0.2	87	2.3 ^y^	23 ^s^	77 ^s^
Cu(20)/MWCNTs [112]	IP	290	0.35 *	300	1	-	-	-	26,700	200	0.1	11	33 ^s^	0 ^s^	62 ^s^
Cu(20)/MWCNTs [112]	IP	290	0.35 *	300	1	-	-	-	26,700	300	0.1	75	63 ^s^	8.5 ^s^	28.5 ^s^
Ni(20)/MWCNTs [17]	IP	271	-	300	1	-	-	-	26,700	200	0.1	7.5	78.5 ^s^	0 ^s^	21.5 ^s^
Ni(20)/MWCNTs[17]	IP	271	-	300	1	-	-	-	26,700	300	0.1	99.7	67.6 ^s^	16.5 ^s^	15.9 ^s^
Au(1)–Cu(20)/MWCNTs [111]	DP	272	-	300	1	-	-	-	26,700	200	0.1	14	29.8 ^s^	0 ^s^	70.2 ^s^
Au(1)–Cu(20)/MWCNTs [111]	DP	272	-	300	1	-	-	-	26,700	300	0.1	83	73 ^s^	8.6 ^s^	18.4 ^s^
Au(1)–Ni(20)/MWCNTs [17]	DP	311	-	300	1	-	-	-	26,700	200	0.1	8	63.2 ^s^	0 ^s^	36.8 ^s^
Au(1)–Ni(20)/MWCNTs [17]	DP	311	-	300	1	-	-	-	26,700	300	0.1	99.8	70.4 ^s^	0 ^s^	29.6 ^s^

*—based on chemisorption measurement; IP—impregnation method, SIP—subsequent impregnation method, CIP—co-impregnation method, CP—co-precipitation method, DP—deposition-precipitation method, UNC—urea nitrate combustion method, S_BET_—specific surface area, y—yield of the product (%), s—selectivity of the product (%), c—concentration of the product (%), m—level in product gas (mol%).

## Abbreviations

ATRM	Autothermal reforming of methanol
CIP	Co-impregnation method
CNTs	Carbon nanotubes
CP	Co-precipitation method
CRM	Combined (oxy–steam) reforming of methanol
DME	Dimethyl ether
DMFC	Direct methanol fuel cell
DP	Deposition–precipitation method
FTIR	Fourier-transform infrared spectroscopy
GHSV	Gas hourly space velocity
IP	Impregnation method
MD	Decomposition of methanol
MWCNTs	Multiwalled carbon nanotubes
OSR	Oxy–steam-reforming of methanol
POM	Partial oxidation of methanol
RDS	Rate-determining step
RWGS	Reverse water gas shift
S_BET_	Specific surface area
SIP	Subsequent impregnation method
SMSI	Strong metal-support interaction
SRM	Steam reforming of methanol
TOM	Total oxidation of methanol
UNC	Urea nitrate combustion method
W/F	Catalyst weight/volume flow rate ratio
WGS	Water-gas shift
XANES	X-ray absorption near edge structure
XPS	X-ray photoelectron spectroscopy
XRD	X-ray diffraction spectroscopy
